# The Institutional Worker

**Published:** 1911-11-25

**Authors:** 


### INSTITUTIONAL WORKER

**Being a Special Supplement to "The Hospital"**

### Editor's Notices.

**Contributions:**

Contributions should be written, or preferably typed, on one side of the paper only, and all articles sent in are accepted upon the distinct understanding that they are forwarded to The Hospital only.

The Editor cannot undertake to return MSS. not used, but when a stamped directed envelope is enclosed the MSS. may be returned if a special request is made.

Accepted articles and paragraphs of news will be paid for after publication at the scale rate.

**Address:**

To prevent delay all contributions and letters on editorial business must be addressed exclusively to the Editor, "The Hospital," 29 Southampton Street, Strand, London, W.C. It is important that this regulation shall be strictly observed.

**Correspondence:**

Correspondence on all subjects is invited. The name and address of correspondents must be given as a guarantee of good faith, but not necessarily for publication.

**Special Articles:**

Special articles are invited, and questions, inquiries, and paragraphs upon all matters relating to the work, administration, and management of general, special, mental, fever, cottage and convalescent hospitals, sanatoria, homes, institutions, societies and organisations for the treatment or care of the sick, injured and dependents of all classes. Every matter affecting the interests and welfare of the staff of all grades working in these institutions will receive special consideration.

**Administrative Medicine, Research and Items of News:**

Special terms will be paid for approved articles on subjects in this wide field by experts who have made some section of it their special study and interest. We wish to give prominence to every movement tending to advance accuracy of diagnosis, efficiency in treatment, and the development of modern methods to eradicate disease and promote the welfare of the suffering.

**A Bureau of Information:**

We invite inquiries and applications for information and help in providing for that numerous class of sufferers who require special care or house-room which they cannot obtain in their own homes or secure for themselves. **We cannot, however, prescribe or recommend practitioners.**

**Books for Review:**

Publishers are particularly requested to send advance proofs of any new books of importance whenever possible, as the Editor has made arrangements to publish immediate reviews on a new plan.

**Photographs, Plans, Blocks, and Illustrations:**

It is requested that wherever possible MSS. may be accompanied by illustrations in any of the above forms. The name of the sender and of the article to which it belongs should be written on the back of each photograph, drawing, or block for purposes of identification.

**Local Papers:**

Newspapers containing reports or news paragraphs **should be marked** and addressed to the Sub-Editor.

**The Coupon System:**

A coupon will be found at the bottom of the third inside page of the cover each week. This coupon must be attached to every question or inquiry to which an answer is desired in The Hospital.

### Manager's Notices.

Letters relating to the publication, sale, and advertising departments of The Hospital must be addressed to the Manager, "The Hospital," The Hospital Building, 28 and 29 Southampton Street, Strand, London, W.C.

**Circulation and Sale:**

The Hospital is on sale at all newsagents and bookstalls every Friday, price one penny. The rates of subscriptions, post free from The Hospital office, have been reduced and are now as follows :—

| | United Kingdom s. d. | The Colonies and Abroad s. d. |
|---|---|---|
| For one year ... ... ... | 6 6 | 8 8 |
| ,, six months ... ... | 4 0 | 5 0 |
| ,, three months ... ... | 2 0 | 2 6 |

**Subscriptions** which may begin at any time, are payable in advance. Cheques and Post-Office orders, crossed London County and Westminster Bank, Covent Garden branch, should be made payable to the Manager of The Hospital and addressed to him at The Hospital Building, 28 and 29 Southampton Street, Strand, London, W.C.

**Advertisements:**

All advertisements for The Hospital must reach the Manager not later than Wednesday morning in each week. For scale of charges see pages v and 2.

**Classified Advertisements:**

All small advertisements will be classified, for this section of the paper is widely read and of special interest. We wish to encourage those wanting appointments who are attracted to institutional work, and therefore offer them the reduced rate of *twenty-one words and under for* 1s., and *for every ten and part of ten words beyond*, 6*d*.

### POINTS FOR INSTITUTION AUTHORITIES.

The economical management of an Institution depends very greatly upon purchasing supplies in the cheapest market.

It is often impossible locally to secure the highest quality at the lowest prices.

In consequence, a section will be reserved in this part of the paper for announcements of Institutions inviting Tenders for Contracts.

This will enable Authorities of Institutions to secure tenders for supplies from all over the country, and ensure purchases at the lowest possible prices consistent with quality.

The charge for Tenders is 6s. per inch, and for this small outlay many pounds may be saved on supplies of all descriptions.

**Special Rates** will be quoted for those institutions which agree to send all their vacancy, contract, and public notices to The Hospital each year, so as to make it a file of such announcements for ready reference.

### POINTS FOR TRADE ADVERTISERS.

The Hospital is the organ of Institutions, whether of a Voluntary Character or those under Local Government, Poor Law and Municipal Authorities.

It is read by all Executive Officials and those responsible for the Construction, Management, and Equipment of Public Establishments.

It is also the Journal of the worker engaged in the Medical, Health and Preventive services.

There is no medium more appropriate and effective in the promotion of business in these directions than The Hospital.

### Situations Vacant.

### Miscellaneous.

SUGGESTED Removal of St. George's Hospital from Hyde Park Corner. Illustrated article by Mr. Mark H. Judge in the *Architect* for November 3rd. Fourpence. (5218)

### APPOINTMENTS VACANT.

### Poor-Law Vacancies.

### Appointments Vacant.

ARCHITECTURAL ASSISTANT (£2 weekly), Bolton Union. Mr. H. I. Cooper, C. to G., 28 Mawdsley Street, Bolton. Dec. 2.

ASSISTANT LAUNDRY SUPERINTENDENT (£23 18s.), St. George-in-the-East Guardians. Mr. R. M. Lochner, C. to G., Raine Street, Old Gravel Lane, E. Nov. 28.

ASSISTANT NURSE (£31), West Ham Union. T. Smith, C. to G., Union Road, Leytonstone, N.E. Dec. 6.

ASSISTANT NURSE (£25), Hartismere Union. H. Warnes, C. to G., Union Offices, Eye, Suffolk. Dec. 1.

ASSISTANT NURSE (£20), King's Lynn Union. Mr. R. C. Coulton, C. to G., Lynn.

ASSISTANT NURSE (£20), Bideford Union. M. J. Durant, C. to G., Bideford. Nov. 27.

ASSISTANT NURSES (£23), Rugby Union. J. W. Pendred, C. to G., Union Offices, Rugby. Dec. 1.

ASSISTANT RATING SURVEYOR (£150), Bolton Union. Mr. H. I. Cooper, C. to G., 28 Mawdsley Street, Bolton. Dec. 2.

BOYS' CARETAKER AND INDOOR LABOUR MASTER (£30), Macclesfield Union. Mr. F. May, C. to G., Macclesfield. Nov. 27.

CASE PAPER CLERK (30s. weekly), Bath Union. Mr. W. E. Winchworth, C. to G., Bath. Nov. 25.

CHARGE DAY NURSE (£30), Guildford Union. W. S. V. Cullerne, C. to G., Commercial Road, Guildford. Dec. 8.

CHARGE NURSE (£35), Hartismere Union. H. Warnes, C. to G., Union Offices, Eye, Suffolk. Dec. 1.

CHARGE NURSE (£28), Brighton Guardians. B. Burfield, C. to G., Parochial Offices, Brighton. Nov. 28.

CHARGE NURSE (£30), West Ham Union. T. Smith, C. to G., Union Road, Leytonstone, N.E. Dec. 6.

CHARGE NURSE (£30), Isle of Wight Union. Mr. A. G. Harrison, C. to G., Newport, I.W. Nov. 29.

CHARGE NURSE (£24), City of London Union. Matron, City of London Infirmary, Clifden Road, N.E.

CHILDREN'S ATTENDANT (£20), Wandsworth Union. Matron, Intermediate Schools, Swaffield Road, Wandsworth.

CLASS SISTER (£50), West Ham Union. T. Smith, C. to G., Union Road, Leytonstone, N.E. Dec. 6.

FEMALE INDUSTRIAL TRAINER (£24), Louth Union. Mr. F. C. Chard, C. to G., Louth. Dec. 2.

FOSTER-MOTHER (£20), Reading Guardians. Mr. W. H. Oliver, C. to G., Reading. Nov. 27.

MALE IMBECILE ATTENDANT (£26), Haslingden Union. Mr. J. H. Sinkinson, C. to G., Union Offices, Rawtenstall. Nov. 27.

MALE LUNATIC ATTENDANT (£30), City of London Union. Medical Officer, 42 Clifden Road, Lower Clapton, N.E.

MALE LUNATIC ATTENDANT (£30), Holborn Union. Master of the Workhouse, 1a Shepherdess Walk, N.

MALE NURSE (23s. weekly), West Ham Union. Mr. T. Smith, C. to G., Leytonstone, N.E. Dec. 6.

MALE NURSE (£25), King's Lynn Union. Mr. R. C. Coulton, C. to G., Lynn.

MASTER AND MATRON (£30 each), Gower Union. Mr. H. J. Ind, C. to G., Fisher Street, Swansea. Nov. 27.

MASTER'S CLERK (£25), Hammersmith Guardians. The C. to G., 206 Goldhawk Road, Shepherd's Bush, W. Nov. 27.

MATRON'S ASSISTANT (£20), Chesterton Union. Mr. J. F. Symonds, C. to G., 9 Bene't Street, Cambridge. Dec. 1.

MIDWIFERY NURSE (£30), Hackney Union. F. R. Coles, C. to G., Clerk's Office, Sidney Road, Homerton, N.E. Nov. 29.

MOTHERS' AND INFANTS' ATTENDANT (£25), Southwark Union. Mr. S. Wood, C. to G., Ufford Street, Blackfriars. Nov. 28.

NURSE (£27 10s.), Stockbridge Union. Matron of the Workhouse, Stockbridge. Dec. 4.

NURSE (£25), Hartley Wintney Union. Mr. W. H. Wright, C. to G., Odiham, Hants.

NURSE (£25), Hay Union. Mr. R. T. Griffiths, C. to G., Hay, Nov. 27.

NURSE (£25), Dursley Union. J. G. Wenden, C. to G., Dursley. Nov. 30.

NURSES (£25), Lewes Union. C. Patrick, C. to G., Union Offices, Lewes. Dec. 7.

NURSERY ATTENDANT (£20), Islington Guardians. Matron of the Workhouse, St. John's Road, Upper Holloway, N.

PROBATIONERS (£10), Wakefield Guardians. H. Beaumont, C. to G., 47 Kirkgate, Wakefield. Nov. 25.

PROBATIONERS (£10), King's Norton Union. Mr. R. J. Curtis, C. to G., Guildhall Buildings, Birmingham.

PROBATIONER NURSE (£10-£20), Haslingden Union. Mr. J. H. Sinkinson, C. to G., Union Offices, Rawtenstall. Nov. 27.

SUPERINTENDENT NURSE (£40), Lambeth Guardians. Mr. J. L. Goldfinch, C. to G., Brook Street, Kennington, S.E. Nov. 27.

Temporary Staff Nurses (3) (£20), Holborn Union. Matron of the Infirmary, Archway Road, Upper Holloway, N.
Ward Sister (£30), Hammersmith Guardians. C. to G., 206 Goldhawk Road, Shepherd's Bush. Dec. 11.
Ward Sisters (2) (£35), Sunderland Union. Mr. W. P. Brantingham, C. to G., Sunderland. Nov. 29.
Workhouse Master's Clerk (£30), Aston Union. Mr. J. North, C. to G., Union Offices, Vauxhall Road, Birmingham. Nov. 27.

### Situations Vacant.

Assistant Engineer and Stoker (37s. weekly), Brentford Union. Mr. W. Stephens, C. to G., Isleworth, W.
Cook and General Assistant (£25), Lymington Union. Mr. J. D. Rawlins, C. to G., Lymington.
Female Cook (£28), Hunslett Union. Mr. F. W. Mee, C. to G., Hunslet, Leeds. Nov. 29.
Gardener and Outdoor Labour Master (25s. weekly, non-resident), Macclesfield Union. Mr. F. May, C. to G., Macclesfield. Nov. 27.
Laundress (£26), Haslingden Union. Mr. J. H. Sinkinson, C. to G., Union Offices, Rawtenstall. Nov. 27.
Laundress (£25), Pontypridd Union. Mr. W. Spickett, C. to G., Pontypridd. Nov. 28.
Laundress (£27), Chelmsford Union. Mr. A. S. Duffield, C. to G., 96 High Street, Chelmsford. Dec. 6.
Laundress (£30), King's Norton Union. Mr. R. J. Curtis, C. to G., Guildhall Buildings, Birmingham.
Laundress (£25), Bosmere and Claydon Union. Matron of the Workhouse, Barham, near Ipswich.
Laundrymaids (£19 and £18), Edmonton Union. Mr. F. Shelton, C. to G., Lower Tottenham.
Master and Matron, Porters, Male Attendants, Cooks, Master's Clerk, Storekeeper, Baker, Engineer, Stokers, Laundry Superintendent, Etc. City of London Union. Mr. E. R. Woodward, C. to G., Bartholomew Close, E.C.

### Institutional and Administrative Vacancies.

### Appointments Vacant.

Charge Nurse (£35), Military Families' Hospital, Bulford. Matron-in-Chief, Queen Alexandra's Imperial Military Nursing Service, War Office, Whitehall, S.W.
Matron (£150), Anglo-American Hospital, Cairo, Egypt. Secretary. Dec. 20. (See advt.)
Matron (£40), Llangollen Cottage Hospital. Hon. Secretary. Nov. 25.
Matron (£40), Stratton Cottage Hospital, N. Cornwall. Hon. Sec., Sanctuary, Stratton. Dec. 6.
Probationers (£10), New Children's Hospital, Sunderland. Apply Matron.
Sisters (£30), New Children's Hospital, Sunderland. Apply Matron.
Staff Nurses (£20), West Ham and Eastern General Hospital. Matron.

### Municipal Vacancies.

### Appointments Vacant.

Assistant Inspectors of Nuisances (5) (£80), Rhondda U.D.C. Mr. W. P. Nicholas, Clerk to the Council, Pentre, Rhondda. Dec. 1.
Assistant Solicitor (£150), Brighton T.C. Mr. H. Talbot, Town Clerk, Brighton. Nov. 27.
Assistant Superintendent of Sewage Pumping Station (£156), West Ham T.C. Mr. F. E. Hilleary, Town Clerk, West Ham. Dec. 2.
Charge Nurse (£30), Hebburn Urban District Council Isolation Hospital. T. Stuart, Clerk to Council. Dec. 2.
Clerk of Works (Schools) (£3 3s. weekly), Leicester T.C. Mr. T. Groves, Secretary for Education, Leicester. Nov. 30.
District Surveyor (£150), North Riding C.C. Mr. W. G. Bryning, County Surveyor, Northallerton. Nov. 30.
General Manager for Tramways and Electricity Department (state salary), Newport T.C. Mr. A. A. Newman, Town Clerk, Newport, Mon. Nov. 27.
Health Visitor (£100), Metropolitan Borough of Poplar. L. Potts, Town Clerk, High Street, Poplar. Dec. 4.
Inspector of Nuisances (£120), Barton-on-Irwell R.D.C. Mr. J. W. Whitworth, Clerk to the Council, Union Offices, Patricroft. Nov. 28.
Inspector of Nuisances (£110), Freebridge Lynn R.D.C. Mr. W. Cross, Clerk to the Council, King's Lynn. Nov. 30.
Land Agent (£250), Lancashire C.C. Mr. H. E. Clare, Clerk to the Council, Preston. Dec. 2.
Matron, Isolation Hospital, Bromsgrove, Worcs.
Nurse (£32 10s.), Lanchester Joint Hospital Board Infectious Hospital. W. H. Ritson, Clerk, Lanchester, Durham. Dec. 4.
Nurse (£25), Lancaster Corporation. Matron, Sanatorium, Lancaster.
Nurses (£19 10s.), London County Asylum, Bexley, Kent. Medical Superintendent.
Nursing Attendants (£18), Metropolitan Asylums Board.
Sisters (£38), Staff Nurses (£26), Assistant Nurses (£24 and £20).
Staff Nurse (£30), Bromsgrove, Droitwich and Redditch Hospital.
Surveyor and Inspector of Nuisances (£120), Barmouth U.D.C. Mr. W. George, Clerk to the Council, Barmouth. Nov. 28.
Surveyor of Highways (£120), Holsworthy R.D.C. Mr. C. Kinsman, Clerk to the Council, Holsworthy. Dec. 5.
Temporary Nurse (£24), Croydon Rural and Merton Joint Hospital Board. Dr. C. M. Fegen, Council Offices, Katharine Street, Croydon.
Town Clerk (£500), Wigan T.C. The Town Clerk, Wigan. Nov. 27.

### Situations Vacant.

Cook (£28-30), Coventry City Hospital. Apply Matron. Dec. 6.
Head Laundress (£40), M.A.B. Matron, Park Hospital, Hither Green.

### Miscellaneous Vacancies.

Assistant Clerk (£52), Essex and Colchester Asylum. Mr. H. H. Gepp, Clerk to the Visitors, Brentwood.
Head Laundress (£34), Newport Mental Hospital. Medical Superintendent of the Hospital, Caerleon, Mon.
Male Cook (£55), Exmouth Training Ship. Captain-Superintendent of the Exmouth, Grays.
Sewing Mistress for Girl's Industrial School (£35), Leeds Education Committee. Mr. J. Graham, Secretary for Education, Leeds.
**School Nurse (£65), Exeter Education Committee. H. Morgan, Clerk, Education Offices, Exeter. Nov. 25.**

### Appointments, Resignations, &c.

POOR LAW APPOINTMENTS.

Bernstein, Mr. B., Fourth Resident Assistant Medical Officer at the Infirmary, West Ham Union.
Gebbie, Mr. N., Resident Assistant Medical Officer at the Infirmary, Salford Union.
Lynch, Mr. J. M., District Medical Officer, Islington Parish.
Porter, Mr. D., Second Resident Assistant Medical Officer at the Workhouse, West Ham Union.
Robertson, Mr. D., Resident Medical Officer at the Workhouse, Prescot Union.
Sutton, Mr. G. F., Assistant Relieving Officer, Hemel Hempstead Union.
Wall, Mr. M. C., Junior Resident Assistant Medical Officer at the Infirmary, Greenwich Union.

